# Dissecting the Roles of Gonadotropin-Inhibitory Hormone in Mammals: Studies Using Pharmacological Tools and Genetically Modified Mouse Models

**DOI:** 10.3389/fendo.2015.00189

**Published:** 2016-01-05

**Authors:** Silvia Leon, Manuel Tena-Sempere

**Affiliations:** ^1^Department of Cell Biology, Physiology and Immunology, University of Córdoba, Córdoba, Spain; ^2^Instituto de Salud Carlos III, CIBER Fisiopatología de la Obesidad y Nutrición, Córdoba, Spain; ^3^Instituto Maimónides de Investigación Biomédica de Córdoba/Hospital Universitario Reina Sofia (IMIBIC/HURS), Córdoba, Spain; ^4^FiDiPro Program, Department of Physiology, University of Turku, Turku, Finland

**Keywords:** GnIH, RFRP, NPFF receptors, RF9, kisspeptin, GnRH, gonadotropins

## Abstract

Reproduction is essential for perpetuation of the species and, hence, is controlled by a sophisticated network of regulatory factors of central and peripheral origin that integrate at the hypothalamic–pituitary–gonadal (HPG) axis. Among the central regulators of reproduction, kisspeptins, as major stimulatory drivers of gonadotropin-releasing hormone (GnRH) neurosecretion, have drawn considerable interest in the last decade. However, the dynamic, if not cyclic (in the female), nature of reproductive function and the potency of kisspeptins and other stimulatory signals of the HPG axis make tenable the existence of counterbalance inhibitory mechanisms, which may keep stimulation at check and would allow adaptation of reproductive maturation and function to different endogenous and environmental conditions. In this context, discovery of the gonadotropin-inhibitory hormone (GnIH) in birds, and its mammalian homolog, RFRP, opened up the exciting possibility that this inhibitory signal might operate centrally to suppress, directly or indirectly, GnRH/gonadotropin secretion, thus reciprocally cooperating with other stimulatory inputs in the dynamic regulation of the reproductive hypothalamic–pituitary unit. After more than 15 years of active research, the role of GnIH/RFRP in the control of the HPG axis has been documented in different species. Yet, important aspects of the physiology of this system, especially regarding its relative importance and actual roles in the control of key facets of reproductive function, remain controversial. In the present work, we aim to provide a critical review of recent developments in this area, with special attention to studies in rodent models, using pharmacological tools and functional genomics. In doing so, we intend to endow the reader with an updated view of what is known (and what is not known) about the physiological role of GnIH/RFRP signaling in the control of mammalian reproduction.

## Introduction

Reproductive maturation (including puberty) and function are indispensable for perpetuation of the species and, thus, are controlled by a sophisticated network of regulatory signals, which impact at the so-called hypothalamic–pituitary–gonadal (HPG) axis (1). In this system, the group of neurons that synthesize and release the decapeptide gonadotropin-releasing hormone (GnRH) acts as the common output pathway through which the brain, and thereby numerous internal and external cues, controls gonadotropins secretion. Accordingly, GnRH neurons are the target of complex regulatory actions, conducted by excitatory and inhibitory signals, which drive – directly or indirectly – the activity of this key neuronal population (1). In recent years, numerous neuropeptides and transmitters with ability to modulate GnRH neurosecretion have been identified. In fact, significant advances have been made in the characterization of stimulatory signals of GnRH neurons. A paradigmatic example is the identification of the puberty/fertility-stimulating neuropeptide, kisspeptin, which has profoundly changed our understanding of how the reproductive brain is controlled and how it interplays with other key neuroendocrine axes (1, 2). However, less progress has been made in the identification of inhibitory signals, which may counterbalance the effects of kisspeptins and other potent elicitors of GnRH/gonadotropin secretion, thereby playing an equally essential role in the precise and dynamic control of the HPG axis.

In this context, identification in 2000 of a novel peptide of the RF-amide superfamily, named gonadotropin-inhibitory hormone (GnIH) on the basis of its action as *Gonadotropin-Inhibitory Hormone*, raised considerable interest (3). For many neuroendocrinologists, this turned into excitement when the putative ortholog of GnIH, encoding the RF-amide peptides, RFRP-1 and RFRP-3, was identified in mammals (4), and the capacity of RFRPs to inhibit gonadotropin release was initially documented in several mammalian species (4–8). These findings paved the way for the characterization of the reproductive (and non-reproductive) roles of GnIH peptides in mammals, including not only their major effects and mode of action in the control of gonadotropin secretion but also their roles on related functions, such as the regulation of food intake (9). In this review, we intend to provide a succinct overview of recent data obtained in preclinical (mostly rodent) models, using pharmacological tools and functional genomics, which help to unveil the physiological relevance of GnIH/RFRP signaling in the regulation of the HPG axis. In addition, the putative role of this system as connecting factor between reproductive function and body energy homeostasis will be briefly discussed.

## Discovery and Major Structural Features of GnIH/RFRP in Mammals

While extensive description of the identification and major features of GnIH and their mammalian counterparts, RFRP, can be found elsewhere in this Special Issue, in this section we will provide, as a means of brief introduction, a succinct recapitulation of key aspects of this class of peptides in mammals, and how they were discovered. Of note, RF-amide peptide superfamily in mammals comprises a number of central regulators of different neuroendocrine axes, including the reproductive (i.e., RFRP and kisspeptins) and lactotropic (i.e., PRL-releasing peptides) axes (9, 10). These peptides share a common RF-amide (Arg-Phe-NH_2_) signature at their carboxyl-terminal region (9). Notably, the first isolation of a RF-amide peptide occurred in an invertebrate species (11), and led to the discovery of a large series of peptides with a similar carboxyl-terminal RF-amide motif in different invertebrate and vertebrate species (9, 10). In this context, in 2000, Tsutsui and colleagues discovered in birds a 12 amino acid hypothalamic neuropeptide, with a Ser-Ile-Lys-Pro-Ser-AlaTyr-Leu-Pro-Leu-Arg-Phe-NH2 sequence, with the distinct capacity to inhibit gonadotropin release by cultured quail pituitaries (3). This was quite a remarkable finding, as no peptide with similar inhibitory activity on gonadotropin secretion had been previously identified in vertebrates; hence, this peptide was named GnIH by its activity as *Gonadotropin-Inhibitory Hormone* (3).

The avian GnIH is produced by a gene encoding a precursor protein of 173 amino acids that gives rise to three peptides after proteolytic cleavage: one is termed GnIH, and the other two are named GnIH-1 and GnIH-2 (12, 13). These peptides have a common carboxyl-terminal LPXRF-amide sequence, where X could be L or Q. In the pro-hormone, these sequences are flanked by glycine residues on the C-terminus, as well by an amidation signal and a basic amino acid on either end, as proteolytic cleavage site (14). Using these features as reference, similar sequences have been investigated in mammals by searching in gene databases. This has allowed the identification of orthologous genes and peptides in a number of mammalian species; some of these sequences/peptides are shown in a Table 1 (*see next page*). Notably, these analyses allowed identification of two perfectly conserved canonical RF-amide peptides, with sequence homology to avian GnIH, in rats, mice, and hamsters, namely RFRP-1 and RFRP-3. Of note, while RFRP-1 displays higher structural homology with avian GnIH than RFRP-3 (4), pharmacological studies strongly suggest that RFRP-3 is likely the functional ortholog of GnIH in mammals (9, 15).

**Table 1:** **RFRP peptides identified in mammals, with bird (quail) sequence provided for comparison**.

Species	Peptide	Sequence	Reference
Mouse	RFRP-1^a^	VPHSAAN**LPLRFa**	(22)
	RFRP-3^a^	NMEAGTRSHFPS**LPQRFa**	
Rat	RFRP-1^a^	VPHSAAN**LPLRFa**	(22)
	RFRP-3	ANMEAGTMSHFPS**LPQRFa**	(16)
Human	RFRP-1	MPHSFAN**LPLRFa**	(22)
	RFRP-3	VPN**LPQRFa**	
Bovine	RFRP-1	SLTFEEVKDWAPKIKMNKPVVNKMPPSAAN**LPLRFa**	(22)
	RFRP-3	AMAHLPLRLGKNREDSLSRWVPN**LPQRFa**	(17)
Ovine	RFRP-1^a^	SLTFEEVKDWGPKIKMNTPAVNKMPPSAAN**LPLRFa**	(6)
	RFRP-3^a^	VPN**LPQRFa**	
Hamster (Siberian)	RFRP-1	SPAPANKVPHSAAN**LPLRFa**	(18)
	RFRP-3	TLSRVPS**LPQRFa**	
Hamster (Syrian)	RFRP-1^a^	SPAPANKVPHSAAN**LPLRFa**	(4)
	RFRP-3^a^	ILSRVPS**LPQRFa**	
Quail	GnIH	SIKPSAY**LPLRFa**	(3)
	GnIH-RP-1^a^	SLNFEEMKDWGSKNFMKVNTPTVNKVPNSVAN**LPLRFa**	(12)
	GnIH-RP-2	SSIQSLLN**LPQRFa**	(12)

*^a^Deduced from cDNA sequence*.

**a**, terminal amide group (characteristic of RF-amide motif).

**Figure 1:**
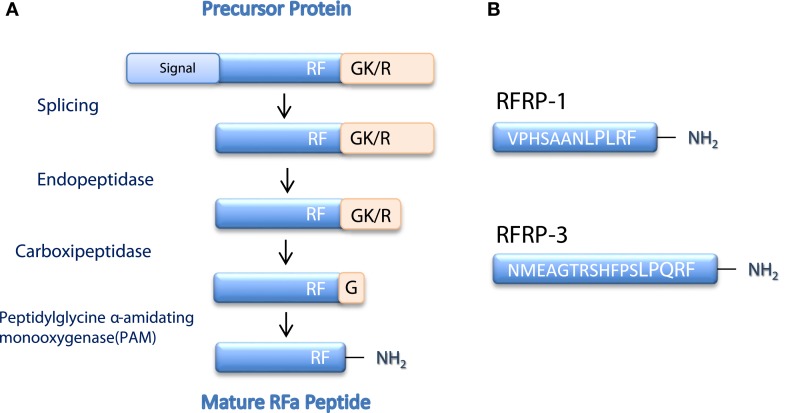
**In (A), a schematic representation of the cascade of proteolytic processing events necessary to generate a RF-amide mature peptide from a precursor protein is shown**. Precursor proteins posses N-terminal regions that contain abundant hydrophobic residues, which form the signal peptide sequence. On the other hand, the precursor proteins contain a RFGR/K motif, in which R/K is a basic amino acid residue and G is an amide donor; cleavage of this area forms the terminal RF-amide signature. In **(B)**, the major structural features and sequences are shown of the two major peptides, RFRP-1 and RFRP-3, encoded by the RFRP gene. For RFRP-1, complete sequence homology is detected between hamster, mouse, and rat sequences. For RFRP-3, the mouse sequence is presented, which is similar to that of the rat peptide, except for one amino acid residue. Based on Ref. (4, 14) with modifications.

Indeed, phylogenetic analyses revealed that in different mammalian species, the RFRP gene encodes two major structurally related peptides: RFRP-1 and RFRP-3 (see Figure 1). These peptides have been identified, among others, in the bovine, rat, mouse, Syrian and Siberian hamster, monkey, and human species (4, 16–21). Additionally, human, macaque, and bovine genes, encoding an LPXRF-amide-like peptide, have been identified and named RFRP-2 (22). Yet, the homolog precursor cDNA in rodents does not encode an equivalent RFRP-2 peptide (10, 23, 24). Moreover, this peptide does not activate the same receptors as RFRP-1 and RFRP-3. Hence, its function as GnIH peptide has been questioned.

The biological actions of RFRP-1 and RFRP-3 peptides are exerted mainly through the G-protein-coupled receptor, NPFF1R (also termed Gpr147). However, RFRP-1 and RFRP-3 can also bind with lower affinity to the related receptor, NPFF2R (also termed Gpr74) (25). In rats, NPFF1R mRNA is expressed in central nervous system, specifically in the hypothalamus, spinal cord, amygdala, hippocampus, and *substantia nigra*, and in peripheral tissues, such as the pituitary gland, gonads, and eyes (22, 26). Furthermore, expression of NPFF1R has been found in the sheep pituitary and human gonadotrophs (20, 27, 28). In the hamster, NPFF1R expression has been observed in all types of germ cells, including sperm, and NPFF2R expression has been detected only in more differentiated germinal cells. In the monkey, RFRPs receptors are found in testicular Leydig cells, spermatogonia, and spermatocytes, as well as in granulosa cells, pre-antral follicles and corpora lutea in monkeys and humans (29).

Regarding neuronal populations expressing RFRPs, these have been detected mainly in the hypothalamic dorsomedial nucleus (DMN) or adjacent areas, and they have been found to project to several hypothalamic regions, including the arcuate nucleus (ARC), the paraventricular nucleus (PVN), the lateral hypothalamus, and the ventromedial nucleus (VMN); all these hypothalamic areas are known to play key roles in the control of reproduction and energy balance (30).

## Pharmacological Analysis of GnIH/RFRP Effects on the HPG Axis in Mammals

On the basis of the original work proposing RFRPs as functional orthologs of GnIH in mammals (4), in recent years a number of studies have aimed to characterize the profiles of (mostly brain) expression, as well as the effects and mechanisms of action of RFRPs on the HPG axis (mainly on gonadotropin secretion) in different mammalian species. For the sake of concision, in this section we will focus on the description of the effects RFRPs agonists, and especially of RFRP-3 as major functional analog of GnIH, on gonadotropin secretion in rodents (mainly) and other mammalian species (see Table 2). In addition, effects of RFRP-3 on related neuroendocrine axes will be also briefly mentioned.

**Table 2:** **Summary of the reported effects of the administration of RFRP-3 in mammals**.

Species	RFRP-3	Reference
Rat	Anti-RFRP ODN treatment in peri-pubertal rats caused a significant increase in plasma LH, but not FSH, levels	(31)
	RFRP-3 administration to peri-pubertal rats caused a significant decrease in plasma LH levels and significant increase in plasma GH levels	
	Central (icv) administration of RFRP-3 significantly reduced plasma LH levels and increased circulating GH, but did not alter plasma levels of FSH, thyroid hormone or cortisol	(5)
	RFRP-3, but not of RFRP-1, analogs had inhibitory effects on LH and/or FSH levels	(15)
	RFRP-3 suppressed the pre-ovulatory GnRH/LH surge independently of the prolactin surge	(32)
	In adult OVX rats, systemic (iv) administration of RFRP-3 lowered plasma LH levels, while icv injections RFRP-3 failed to alter neither the mean levels of LH nor the frequency of the pulsatile LH secretion	(5)
	In cultured pituitary cells, the suppressive effect of RFRP-3 on LH secretion was not clearly detectable in the absence of GnRH, but in the presence of GnRH, RFRP-3 significantly suppressed LH secretion *in vitro*	
	RFRP-3 administration to OVX rats had no detectable effects on basal LH secretion, but inhibited GnRH-stimulated LH secretion	(34)
	Icv injection of RFRPs increased the plasma levels of ACTH and oxytocin	(35)
	In adult males, icv injection of RFRP-3 significantly reduced plasma levels of LH and increased GH independently of the time of day; however, it did not alter plasma FSH, thyroid hormone, or cortisol levels	(8)
Hamster	In female (OVX) Syrian hamsters, GnIH administration (central or peripheral) rapidly inhibits LH secretion	(7)
	In male Syrian hamsters, acute central injection of RFRP-3 induces c-Fos expression in GnRH neurons and increases LH, FSH, and testosterone secretion	(36)
	Central (icv) administration of RFRP-1 or RFRP-3 to male Siberian hamsters decreased LH and FSH levels in long-day conditions, but stimulated LH secretion in short-day conditions	(18)
Mouse	In electrophysiological studies, an inhibitory effect of RFRP-3 on the excitability of GnRH neurons has been shown in male and female mice	(37)
	RFRP-3 has been shown to lower plasma LH levels *in vivo* (after icv injection) and LH secretion *in vitro* (pituitary explants)	(38)
	RFRP-3 has been suggested to inhibit testicular steroidogenesis and spermatogenesis, either indirectly through GnRH or by directly influencing germ cell proliferation, survival, and apoptosis	(39)
	GnIH inhibited follicular development and steroidogenesis in the mouse ovary	(40)
Sheep	Peripheral administration of GnIH decreased the amplitude of LH pulses, while GnIH decreased the secretion of LH and FSH *in vitro*	(6)
Human	RFRP-3 reduced FSH, LH, and forskolin-stimulated progesterone secretion by human granulosa cells	(41)

### Effects of the Administration of RFRP-3 in Rats

The limited (indirect) data available to date seem to indicate that in the rat the GnIH/RFRP system does not play an essential role in the regulation of the HPG axis during the developmental period that takes place before puberty. This is based on the fact that central administration of the agonist, RFRP-3, or antisense oligonucleotides (ODNs) against RFRP-3, did not alter the timing of puberty in male rats (31). However, RFRP-3 infusion elicited a significant decrease in luteinizing hormone (LH) levels, while it significantly increased plasma growth hormone (GH) levels compared to control rats, suggesting that RFRP-3 may be associated with peri-pubertal rise in GH secretion (31).

In turn, the GnIH/RFRP system seems to participate in both sexes in the maintenance of basal levels of gonadotropins in adulthood, exerting an inhibitory effect on the HPG axis, which is evidenced by the fact that central administration of RFRP-3 or its analog, RFRP3–8, induces a rapid decrease in serum LH levels (15). Similarly, central injection of RFRP-3 decreased the magnitude of the pre-ovulatory surge of LH, independently of the prolactin surge (32). The inhibitory effects of RFRP-3 were more clearly detected in gonadectomized (GNX) rats, used as putative model to ease identification of potential inhibitory effects against the prevailing elevated levels of gonadotropins caused by GNX. These analyses revealed consistent, albeit modest, inhibitory effects of RFRP-3, but not of RFRP-1, analogs on LH and follicle-stimulating hormone (FSH) levels, especially at high doses (15). As mentioned earlier, it is intriguing that RFRP-1 has been shown to display a higher degree of homology with the avian GnIH than RFRP-3 (25). Yet, the pharmacological analyses in rats strongly suggest that RFRP-3 is actually the functional homolog of GnIH in mammals (15). In fact, studies in ovine and bovine species have confirmed inhibitory effects of RFRP3–8 on gonadotropin secretion by cultured pituitary cells (6, 33), with higher bio-potency than the larger 17-amino acid RFRP-3 fragment. Moreover, the capacity of central injection of RFRP3–8 to inhibit LH secretion has been documented not only in GNX female rats but also in intact and GNX male rats (15). Inhibitory actions of centrally injected RFRP-3 have been also reported on FSH secretion in male rats *in vivo*. Although unambiguous, the magnitude of such responses was somewhat modest and detected only at the high dose range (≥1 nmol/rat) (15).

In rats, the ability of RFRP to inhibit gonadotropin secretion directly at the pituitary gland remains controversial. In fact, some studies failed to detect GnIH/RFRP-immunoreactive fibers in the median eminence (34). Yet, expression of NPFF1R has been detected in the pituitary gland. In line with the latter, our pharmacological experiments supported a direct action of GnIH/RFRP at the pituitary in rats, as the octapeptide fragment of RFRP-3 was able to decrease basal and GnRH-stimulated secretion of LH by pituitaries from GNX males *ex vivo* (15). Interestingly, comparison of the effective dose-window revealed a dominant inhibitory action of RFRP-3 on GnRH-stimulated LH secretion at the low physiologic range (10^−10^ M), which is lost at higher concentrations (10^−6^ M) of the neuropeptide (15).

Additional studies have addressed the predominant (hypothalamic vs. pituitary) site of action of RFRP-3 in the inhibitory control of the HPG axis in rats by a combination of *in vivo* and *in vitro* approaches. Thus, comparison of the effects on LH secretion of central [intra-cerebroventricular (icv)] vs. systemic [intravenous (iv)] administration of RFRP-3 in adult ovariectomized (OVX) female rats revealed that, while iv administration of RFRP-3 significantly reduced plasma LH levels, icv RFRP-3 injections failed to alter neither the mean LH levels nor the ­frequency of the pulsatile LH secretion (8). The latter is at odds with other studies addressing the effects of central administration of RFRP-3, as described above, and points to a predominant pituitary action of RFRP-3. In the same vein, studies using cultured pituitary cells from female rats demonstrated a suppressive effect of RFRP-3 on LH secretion, selectively in the presence of GnRH (8). Alike, another study showed that RFRP-3 administration to OVX rats had no effects on basal secretion, but it inhibited GnRH-stimulated LH secretion by about 25% (34).

As final comment to this section, it has been documented that in adult males, icv injection of RFRP-3 has been shown to significantly increase GH secretion, independently of the time of day, while it did not alter plasma levels of thyroid hormone, or cortisol (5). In addition, RFRP-3 also has been related to the control of neuroendocrine and behavioral stress responses in rats. In this context, icv injection of RFRP-3 increased the expression of Fos protein in oxytocin neurons in the hypothalamus and plasma levels of adrenocorticotropic hormone and oxytocin (35).

### Effects of the Administration of RFRP-3 in Hamsters

In hamsters, the role of the GnIH/RFRP system in the maintenance of basal levels of gonadotropins was initially suggested by the observation that in OVX Syrian hamsters, icv injection of GnIH induced a rapid reduction of LH levels; similar results were obtained after peripheral injections of the peptide (4). By contrast, however, a somewhat paradoxical stimulatory role of GnIH/RFRP on the gonadotropic axis has been demonstrated in male Syrian hamsters. Thus, acute central injection of RFRP-3 has been shown to induce c-Fos expression in GnRH neurons, and to increase LH, FSH, and testosterone secretion in male (but not female) Syrian hamsters (36). This result suggests that the effects of RFRP-3 administration on the gonadotrophic axis may be sex dependent, at least in the Syrian hamster (36). Of note, icv administration of RFRP-1 or RFRP-3 to male Siberian hamsters induced a significant decrease in LH and FSH levels under long-day photoperiodic conditions, while, in sharp contrast, both peptides stimulated LH secretion after administration to hamsters under a short-day regimen (18). This would suggest that, in addition to sex, external light cues and photoperiod would play a dominant role in defining inhibitory vs. stimulatory responses to GnIH/RFRP in hamsters.

### Effects of the Administration of RFRP-3 in Mice

Electrophysiological studies have shown an inhibitory effect of RFRP-3 on the excitability of GnRH neurons in male and female mice (37). The effects were found to be of rapid onset, dose dependent, and repeatable, suggesting a typical neurotransmitter mode of action of RFRP-3 on GnRH neurons. However, it is noted that RFRP-3 displayed mixed actions on the firing rate of GnRH neurons, with >40% being inhibited but 12% being activated by RFRP-3 (37). In any event, these observations as a whole support a role for RFRP-3 in the modulation of GnRH neuron activity, as major mechanisms for its action in the regulation of gonadotropin secretion (37). To date, the effects of RFRP-3 on gonadotropin secretion *in vivo* have been scarcely studied in the mouse. In fact, to our knowledge, only one study, coming from our group, has shown an inhibitory effect of RFRP-3 on LH secretion *in vivo* in the mouse, using a standard OVX model. Thus, central (icv) administration of 5 nmol RFRP-3 to OVX mice evoked a 25% suppression of LH levels (38). Likewise, pituitary explants from male mice, exposed to RFRP-3 *in vitro*, displayed a suppression of LH secretion (38). In addition, a recent study showed that RFRP-3 treatment induces significant changes in body mass, circulating steroid level and testicular activity in mice (39). Notably, RFRP-3 treatment also caused dose-dependent histological changes in spermatogenesis, such as a decline in germ cell proliferation and survival markers and an increase in apoptotic markers in testis. This study also suggested that the inhibitory effect of RFRP-3 in the testis might be mediated through local production of GnRH. Thus, RFRP-3 could inhibit testicular steroidogenesis and spermatogenesis either indirectly through GnRH or by directly influencing germ cell proliferation, survival, and apoptosis (39). Additionally, GnIH has been shown to inhibit follicular development and steroidogenesis in the mouse ovary (40).

### Effects of the Administration of RFRP-3 in Ovine and Primate Species

While, for sake of concision, we have focused our review in rodent studies, it is interesting to note that *in vivo* and *in vitro* studies have demonstrated an inhibitory effect of GnIH on reproduction in ewes, where the peripheral administration of GnIH decreased the amplitude of LH pulses, while *in vitro* GnIH decreased the secretion of LH and FSH (6).

In addition, it is notable that the RFRP/NPFF1R system has been shown to be expressed in the gonads of primates; thus, indirectly suggesting that GnIH/RFRP might exert a direct role in the control of gonadal physiology. In this sense, treatment of human granulosa-lutein cells with RFRP-3 reduced FSH, LH, and forskolin-stimulated progesterone secretion (41). To our knowledge, the effects of GnIH/RFRP on the secretory profiles of gonadotropins in primates (including humans) have not been reported to date.

## Analysis of GnIH/RFRP Roles on the HPG Axis Using Antagonists: Studies with RF9

As summarized in previous sections, the available pharmacological data strongly suggest that RFRPs are involved in the regulation of the HPG axis in mammals, by delivering (predominantly) an inhibitory signal to the central elements of the gonadotropic axis. However, as also reviewed above, the integral analysis of the pharmacological data surfaces some inconsistencies and differences, which have fueled the debate on the actual physiological relevance, relative importance (vs. other regulatory systems) and major sites of action of this neuropeptide system in the control of gonadotropin secretion in mammals (25). To some extent, this is due to the fact that the study of the physiological actions of GnIH/RFRP has been mostly based on the use of indirect experimental approaches, involving expression analyses or the testing of pharmacological doses of exogenous agonists. By contrast, direct assessment of the roles of endogenous RFRPs in mammals has been hampered by the lack of potent and selective antagonists of RFRP signaling.

In this context, in 2006, the compound RF9 was reported as a potent and selective antagonist of NPFF receptors, with binding affinity and antagonistic activity at the level of both NPFF1R and NPFF2R (42). Notably, initial pharmacological characterization of this compound focused on the analyses of NPFF-mediated events; it was shown that RF9 effectively blocks the effects of NPFF on heart rate and blood pressure, and it was capable to prevent opioid-induced hyperalgesia and tolerance in rats, phenomena that are presumably mediated via NPFF2R (42). However, it was not until 2010 when the first study addressing the impact of RF9-mediated RFRPR blockade, as putative receptor pathway for mediating GnIH/RFRP actions, on gonadotropin secretion in rodents was published by our group (43).

The effects of RF9 on gonadotropin secretion were initially explored in cycling female rats. Intracerebral injection of RF9 evoked robust LH secretory responses in cycling rats at the two stages of the cycle tested, estrus and diestrus-1 (D-1), with peak values at 30 min and persistently elevated LH levels during the 120-min period after RF9 injection. The testing of a wide range of doses of RF9 (10 and 100 pmol, and 1, 5, and 20 nmol; icv injection) demonstrated consistent stimulation for doses of RF9 from 5 nmol onwards (43). Similar dose–response curves were generated in adult male rats after icv injection of different doses of RF9. Consistent stimulation of LH secretion was detected in male rats from doses of 1 nmol/icv onward. In addition, RF9 elicited significant elevations of circulating FSH levels in cycling females at estrus, but not at D-1; moreover, only the dose of 20 nmol RF9 icv was capable of eliciting unambiguous FSH responses in male rats (43).

In mice, RF9 elicited robust LH secretory peaks at 15 min after its icv injection, which represented >20-fold increase over basal levels; LH responses to RF9 in mice were similar in amplitude between males and females (43, 44). However, central injection of RF9 failed to evoke significant FSH responses in female mice, whereas it elicited a modest 35% increase in serum FSH levels in adult males. In addition, RF9 blocked the inhibitory effects of NPFF on GnRH neuron pacemaker activity (45).

The potential interplay of GnIH/RFRP with sex steroid levels and the functional status of the HPG axis in the control of gonadotropin secretion have been pharmacologically explored also in various species using RF9. Thus, iv administration of RF9 increased plasma LH levels in orchidectomized (ORX) male rats, but this stimulatory effect was completely blunted after blockade of GnRH actions (46). On the other hand, the stimulatory effects of RF9 on gonadotropin secretion were detected despite the prevailing suppression of gonadotropin levels by testosterone or estradiol. In fact, central administration of RF9-induced extraordinarily potent *in vivo* LH responses in ORX mice receiving a fixed dose of testosterone, while RF9 reversed the inhibitory effects of testosterone on GnRH release frequency from brain slices in vitro (47). In turn, blockade of estrogen receptor-α partially attenuated gonadotropin responses to RF9 in rats (43). In other species, like the ewe, icv injection of RF9 during anestrus or the breeding season caused a clear elevation of plasma LH levels, with a more pronounced effect during the anestrous season (48). Furthermore, peripheral administration of RF9 as a bolus or as a constant iv infusion to anestrous ewes induced a sustained increase in plasma LH levels (48). A summary of the reported effects of RF9 in different mammalian species is presented in Table 3.

**Table 3:** **Summary of the reported effects of RF9 administration on the HPG axis in mammals**.

Species	RF9	Reference
Rat	Intravenous treatment with RF9 increased circulating LH levels in ORX male rats, but failed to evoke LH secretion after blockade of GnRH actions	(42)
	Central administration of RF9 evoked a dose-dependent increase of LH and FSH levels in adult male and female rats	(43)
	Administration of RF9 further augmented the gonadotropin-releasing effects of kisspeptin (duration of responses), and its stimulatory effects were detected despite prevailing suppression of gonadotropin secretion by testosterone or estradiol	
	In males, systemic administration of RF9 modestly stimulated LH secretion *in vivo* and had no direct effects in terms of gonadotropin secretion by the pituitary *in vitro*	
	Co-administration of the kisspeptin antagonist, p234, blunted LH responses to RF9	(44)
Mouse	Central (icv) injection of RF9 elicited with robust LH secretory responses in mice	(43)
	LH responses to RF9 were severely blunted in Gpr54 KO mice, with absolute magnitudes that were only one-tenth of WT mice	(44)
	Central (icv) administration of RF9 induced potent LH responses in ORX mice getting a fixed dose of testosterone, but these were absent in ORX Gpr54 KO mice with similar testosterone replacement	
	RF9 blocked the inhibitory effects of NPFF on GnRH neuron pacemaker activity and reversed the inhibitory effects of testosterone on GnRH secretory frequency	(45)
	Central (icv) administration of RF9 evoked very potent LH secretory responses in mice genetically devoid of NPFF1R	(51)
Sheep	Central (icv) and peripheral administration of RF9 induced significant increases in LH plasma concentrations in the ewe, especially in the anestrous season	(48)

Despite the fact that the above pharmacological evidence is suggestive of a tonic suppression of gonadotropin tone by GnIH/RFRP, as blockade of this signaling pathway by RF9 causes a robust increase in LH (and to a lesser extent, FSH) levels, recent doubts have been raised about the interpretation of hormonal studies using this GnIH/RFRP antagonist. A call of caution for interpretation of the gonadotropic effects of RF9 was made already in initial publications, given the capacity of RF9 to block both NPFF1R and NPFF2R. Yet, the function of RF9, as putative selective antagonist of NPFFRs, together with the conspicuous lack of other NPFF1R antagonist, made RF9 an appealing candidate for neuroendocrine studies.

These doubts have recently substantiated in the context of analyses of the potential interplay of GnIH/RFRP and kisspeptins using RF9 as pharmacological probe. Initial studies demonstrated that co-injection of RF9 and kisspeptin-10 (Kp-10) resulted in elevated LH levels that were similar to those observed after icv administration of RF9 alone, except for a longer duration of those evoked by the combined administration of RF9 and Kp-10 (43). In fact, co-administration of RF9 and Kp-10 elicited FSH secretory responses that were not statistically different from those of RF9. To further explore the putative interplay between GnIH/RFRP and kisspeptins, the effects of RF9 on gonadotropin secretion were evaluated in Gpr54 KO mice, which are genetically engineered to lack kisspeptin signaling. While persistent LH responses to RF9 were observed in the absence of functional kisspeptin receptors, the absolute magnitude of such responses was severely blunted, as it was about one-tenth of that observed in WT animals (44). Moreover, LH responses to RF9 were totally suppressed in ORX Gpr54 KO mice receiving testosterone implants (44). Altogether, these data strongly suggest that a preserved kisspeptin signaling is essential for the manifestation of the potent gonadotropin-releasing effects of RF9. Along with this view, it was recently demonstrated that the excitatory effects of RF9 on GnRH neuronal firing do not occur in Gpr54 KO mice (49). Furthermore, it has been very recently shown that pharmacological blockade of kisspeptin receptors, by the use of the antagonist p234, blunted RF9-induced LH secretion in female rats (50). Admittedly, however, these observations do not necessarily exclude the possibility that the lowering of inhibitory actions of GnIH/RFRP, eventually caused by RF9, might need preserved kisspeptin inputs to translate into detectable LH secretory responses.

Yet, very recent studies, combining *in vitro* and *in vivo* analyses, further support a primary action of RF9 directly via Gpr54 (51). Thus, analyses using CHO cells stably expressing Gpr54 have demonstrated the capacity of RF9 to bind the kisspeptin receptor and to activate its canonical intracellular signaling cascade, including increases in intracellular calcium and inositol phosphate, as well as ERK phosphorylation (51). In good agreement, our studies *in vivo* have demonstrated that RF9 evokes very potent LH-releasing responses in a mouse line genetically devoid of NPFF1R, the Npff1r^−/−^ mouse (see Analysis of the GnIH/RFRP Roles in the HPG Axis Using Functional Genomics: *the Npff1r^−/−^Mouse*). Moreover, while LH responses to RF9 were severely blunted in Gpr54 null mice, the stimulatory effects of RF9 were rescued by selective re-expression of Gpr54 in GnRH neurons (51). Altogether, these observations strongly suggest that at least a substantial component of the secretory effects of RF9 on gonadotropin secretion stems from its capacity to activate Gpr54, rather than its blocking effects on NPFF1R.

As final note to this section, a very recent study has reported the identification and pharmacological characterization of a novel NPFFR antagonist, termed GJ14, with improved specificity in terms of receptor interaction and blockade (52). Notably, this study has documented that infusion of GJ14 effectively blocked the anxiogenic and corticosterone-stimulatory effects of RFRP-3 in mice (52). To our knowledge, the impact of this novel antagonist on the function of the HPG axis has not been reported so far. Similarly, additional *in vivo* studies are warranted to further evaluate the specificity of GJ14 and its eventual (lack of) interaction with other central regulators of GnRH/gonadotropin secretion.

## Analysis of the GnIH/RFRP Roles in the HPG Axis Using Functional Genomics: *The Npff1r^**−/−^*MOUSE**

As summarized in previous sections, compelling pharmacological evidence suggests that GnIH/RFRP plays a role in the inhibitory control of the HPG axis in mammals. However, the actual physiological relevance of this system remained incompletely defined, mainly because of conflictive results concerning the nature, magnitude, and major sites of action of RFRP-3 in the control of gonadotropin secretion. To some extent, these uncertainties were due to the lack of appropriate experimental models and investigative tools to address the physiological functions of GnIH/RFRP *in vivo*. A major advancement in this area, however, took place recently, when the reproductive characterization of the first mouse line with genetic inactivation of NPFF1R was published by our group (38). This piece of work, which analyzed the impact of congenital ablation of NPFF1R on fecundity, litter size, puberty, adult gonadotropic function, gonadal feedback, and NPFF/Kiss1 interactions, is considered relevant to ascertain the relative importance of this system in the control of reproductive function in mammals (38).

As expected, mice deficient for NPFF1R did not respond to icv administration of RFRP-3 (38), in contrast to the preserved inhibitory responses observed in WT mice, where RFRP-3 suppressed LH secretion, in keeping with previous literature (15). In addition, while pituitary explants from WT male mice responded to RFRP-3 with a significant suppression of LH secretion *in vitro*, this inhibitory response was not detected in the pituitaries from Npff1r^−/−^ animals (38). The latter data evidence that RFRP-3 can partially act directly at the pituitary level to suppress LH secretion in mice. Interestingly, whereas pituitaries from both WT and NPFF1R KO mice responded to GnRH with robust LH secretory responses *in vitro*, the magnitude of such responses was (modestly) higher in NPFF1R KO mice, suggesting that null animals are devoid of inhibitory mechanisms that restrain stimulated LH secretion (38).

In keeping with the presumable role of GnIH/RFRP as inhibitor of gonadotropin secretion, NPFF1R-deficient male and female mice had preserved fertility. In fact, the mean size of litters from NPFF1R KO pairs was significantly higher than that of WT breeders (38). Pubertal analyses of null animals evidenced that KO males, but not females, displayed constitutively elevated LH levels before and during puberty, whereas FSH levels were similar between genotypes during the pubertal transition. Pubertal progression was not apparently altered by the congenital lack of NPFF1R, as evidenced by similar mean ages of occurrence of external signs of puberty: balano-preputial separation in males and vaginal opening in females. In addition, testicular and ovarian maturation, as well as ovulatory dynamics, were similar in WT and KO mice, and NPFF1R KO female mice showed preserved estrous cyclicity and pre-ovulatory LH surges of similar magnitude as in WT mice (38).

In addition, the functionality of the gonadotropic axis in the absence of GnIH/RFRP signaling was further explored by studying LH responses to three major regulators of gonadotropin secretion, namely GnRH, Kp-10, and senktide, in NPFF1R null male mice. Of note, senktide is an agonist of neurokinin B (NKB), which has been shown to be co-expressed in a subset of Kiss1 neurons, located in the ARC, where NKB would operate as activator of kisspeptin release onto GnRH neurons (2). Systemic [intra-peritoneal (ip)] injection of GnRH elicited enhanced LH responses in KO mice, in good agreement with the responses observed in the incubations of pituitary explants. In turn, central administration of Kp-10 induced an increase in LH levels similar in both genotypes, while activation of NKB signaling induced lower LH responses in NPFF1R KO mice compared to WT animals. Such differences in the patterns of response to these factors (GnRH, Kp-10, NKB) are probably due to the differences in their sites of action as GnRH acts at the pituitary whereas NKB operates mainly at the level of ARC Kiss1 neurons, and how these sites are differentially affected by the lack of GnIH/RFRP actions (38). In addition, the analysis by *in situ* hybridization of the levels of Kiss1 mRNA in the two major hypothalamic areas of expression (the ARC and the anteroventral periventricular nucleus, i.e., AVPV) in NPFF1R KO mice showed that the overall expression level of Kiss1 and the number of Kiss1 neurons were significantly higher in the ARC, but not in the AVPV, of NPFF1R KO animals, suggesting that RFRP signaling carries out a tonic repression of Kiss1 gene expression at this site (38). Of note, it has been reported that approximately one-forth of ARC Kiss1 neurons actually co-express NPFF1R, while only a very small fraction of AVPV Kiss1 neurons expressed NPFF1R or NPFF2R (53).

The putative interplay between GnIH/RFRP and kisspeptin was further explored by the generation of a double KO mouse, where both inhibitory (NPFF1R) and stimulatory (Gpr54) receptors were genetically ablated. With this approach, we aimed to explore whether elimination of the inhibitory signal driven by RFRP might (at least partially) compensate the profound hypogonadotropic hypogonadism induced by the lack of kisspeptin signaling. However, analysis of this double NPFF1R/Gpr54 KO mouse line revealed that the absence of RFRP signaling was not sufficient to rescue nor did it improve the severe gonadal failure of Gpr54 KO mice, as this double mutant line showed remarkable phenotypic and hormonal similarities with single Gpr54 null mice (38). These findings strongly suggest that, despite some interplay between kisspeptin and GnIH/RFRP signaling, kisspeptin function clearly predominates in the central control of the HPG axis.

Finally, the roles of GnIH/RFRP in the negative feedback control of gonadotropin secretion were also explored using Npff1r^−/−^ mice. To this end, protocols of GNX were applied to male and female WT and KO mice. Removal of sex steroids revealed a delay in LH responses to the removal of gonadal steroids in the mutant mice of both sexes. However, in longer term, the absence of the NPFF1R caused sexually dimorphic alterations in post-GNX responses: while in female mutant mice, circulating levels of LH were significantly higher than those in WT from 7-days post OVX onward; in male mice, LH responses were normalized 7-days after ORX, and remained similar to those of WT from that period onward. These data suggest that in females, but apparently not in males, the GnIH/RFRP system operates as (moderate) brake to modulate the increase in circulating LH levels following the removal of negative feedback signals from the gonads (38).

In sum, functional genomic approaches have allowed to characterize the neuroendocrine (gonadotropic) impact of the constitutive lack of GnIH/RFRP signaling, thereby providing a direct assessment of the relative importance of this system in the control of the HPG axis. These analyses revealed alterations, such as increased litter size, increased gonadotropin levels at certain developmental stages, increased Kiss1 expression in the ARC, and altered post-OXV responses in the Npff1r^−/−^ mouse, which are in general compatible with an inhibitory role of GnIH/RFRP signaling in the control of reproductive function. It must be stressed, however, that most of these changes were moderate in nature and subordinated to preserved kisspeptin signaling (38). Yet, the possibility exists that developmental compensation (e.g., via NPFF2R, whose expression is preserved in this KO model) might have masked to some extent the phenotypic impact of the lack of NPFF1R signaling.

## GnIH/RFRP as Putative Link for the Integral Control of Reproduction and Metabolism

The existence of a close relationship between energy balance and reproduction is well established and there is compelling evidence that forms of metabolic stress, such as conditions of negative energy balance, result in the inhibition of reproductive function. Similarly, alterations of the HPG axis may be linked to metabolic perturbations (54). Despite this evidence, the basic mechanism by which this reciprocal control occurs remains partially unknown. This has prompted the analysis of the metabolic effects of different factors primarily involved in the control of reproductive function and vice versa.

In this context, evidence has been recently presented supporting a potential role of GnIH/RFRP as a potential link between reproductive and metabolic homeostasis. Thus, central (icv) administration of RFRP-3 has been shown to stimulate food intake in adult male rats (55). In turn, it has been reported that RF9 decreased food intake without affecting body weight in female rats (50); yet, the possibility exists that RF9 might evoke such anorectic responses acting via Gpr54, as kisspeptin has been shown to suppress feeding as well (56). However, microinjections of RFRP-3 in the central part of amygdala resulted in a significant decrease of food intake in rats; an effect that was eliminated after RF9 pre-treatment (57). All in all, the pharmacological data available suggest a predominant orexigenic role of RFRP-3, supported also by data obtained in sheep (55); yet, at some brain areas, GnIH/RFRP signaling might evoke food-suppressing responses. Assuming that orexigenic factors are activated in conditions of negative energy balance (as to promote food seeking), it is tenable that GnIH/RFRP might contribute to the suppression of gonadotropic function observed in condition of energy deficit (55).

This possibility has been recently addressed using the Npff1r^−/−^ mouse and protocols of short-term fasting as form of metabolic stress (38). While in WT animals, food deprivation caused a significant lowering of LH levels already at 12-h after beginning of fasting, such a rapid drop of LH was not detected in NPFF1R KO mice subjected to a similar fasting regimen, despite the lowering of body weight was proportionally higher than that in WT animals (38). This observation suggests that activation of GnIH/RFRP signaling might contribute to the suppression of gonadotropin levels observed in conditions of negative energy balance. Indeed, previous studies have shown an increase in the activity of RFRP-positive neurons during periods of undernutrition in hamsters (58). Interestingly, NPFF1R null mice displayed also partially preserved gonadotropic function in response to other forms of metabolic stress linked to reproductive alterations, such as diet-induced obesity. Thus, feeding with high-fat diet (HFD) for 9 weeks evoked >40% weight gain in WT mice, which was associated with a significant decrease in circulating LH levels. By contrast, in NPFF1R KO mice, HFD feeding did not induce significant changes in circulating LH levels, in spite of a similar weight gain as in WT mice (38). Overall, these observations suggest that the mechanisms by which extreme metabolic conditions inhibit gonadotropin secretion probably involve changes in signaling by GnIH/RFRP, such as an increase of the inhibitory tone by RFRP.

## Concluding Remarks

**Figure 2:**
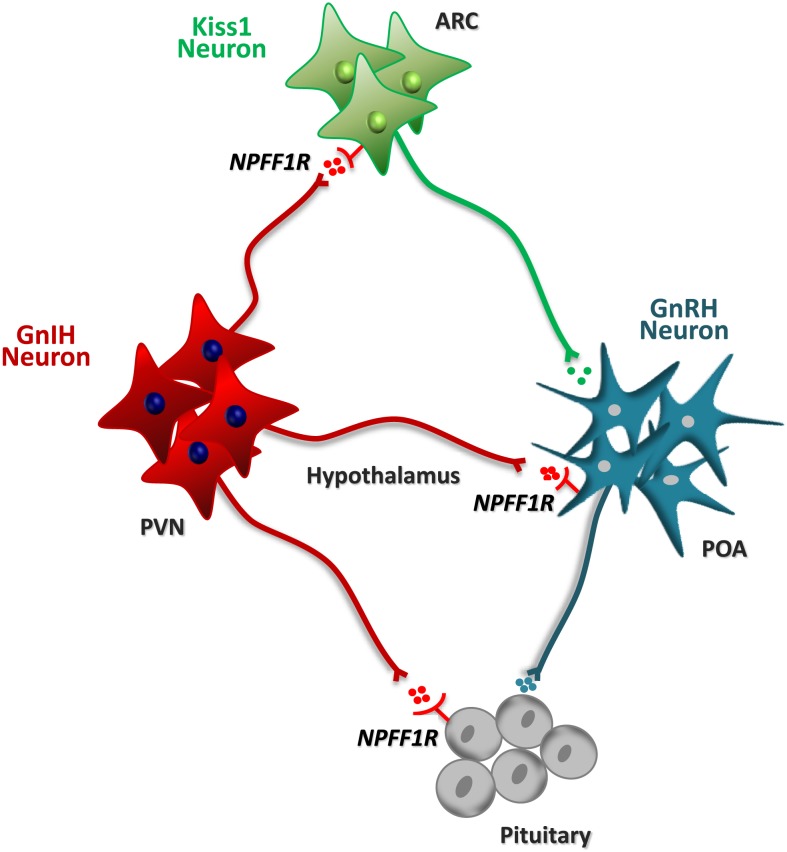
**Schematic diagram of the putative interplay of neurons expressing GnIH/RFRP with key elements of the HPG axis, including pituitary gonadotrophs and hypothalamic GnRH and Kiss1 neurons**. A prominent population of GnIH neurons has been identified in the paraventricular nucleus (PVN). On the basis of neuroanatomical studies (e.g., on neuronal projections) and expression analyses (e.g., of receptors), different sites of action of the GnIH/RFRP system have been proposed, as depicted in the scheme. Admittedly, however, the relative importance of the different sites of action might vary between mammalian species. Alike, direct gonadal effects of GnIH/RFRP (not depicted in this scheme) have been proposed also on the basis of experimental data.

Identification of GnIH in birds, and of its mammalian orthologs, RFRPs, has opened up new avenues for our understanding of the central mechanisms for the control of the HPG axis. As illustrated in Figure 2, expression and functional studies strongly suggest that GnIH/RFRP signaling may play a role in the dynamic control of key elements of the HPG axis, including prominently pituitary gonadotrophs and hypothalamic GnRH and (eventually) Kiss1 neurons. However, while the pharmacological data gathered to date point to a predominant inhibitory effect of GnIH/RFRP on gonadotropin secretion across mammals, some controversy persists regarding the nature (stimulatory in some instances), major sites of action (hypothalamic vs. pituitary) and relative importance (as compared with other neuropeptides) of the GnIH/RFRP system in the control of the gonadotropic axis in different species. Alike, the interplay of RFRP with other central transmitters with key roles in the reproductive brain, such as kisspeptins, has been suggested, but further investigation is needed to fully expose the interactive partners and interdependence of GnIH/RFRP with key central and peripheral regulators of the HPG axis. In this review, we intended to provide a succinct view of the state-of-the-art of the field, by summarizing recent pharmacological data and studies using genetically modified models. By doing so, we aimed to define what we know, and we do not know, about the physiology of GnIH in mammals, as a means to set the scene for further research in this exciting and rapidly evolving area of Neuroendocrinology.

## Author Contributions

In collaboration with the other co-author, this author reviewed the literature, wrote the review, and prepared/edited the Tables and Figures.

## Conflict of Interest Statement

The authors declare that the research was conducted in the absence of any commercial or financial relationships that could be construed as a potential conflict of interest.
